# Supplementary material for: From sequence to enzyme mechanism using multi-label machine learning
Source: BMC Bioinformatics. 2014 May 19;15:150. doi: 10.1186/1471-2105-15-150 (PMC4229970; doi:10.1186/1471-2105-15-150)
Supplement: Additional file 2 — Java code of ml2db. Additional file ml2db_code.tar.gz contains the Java source code to run the multi-label machine learning experiments and save the results to database. The code’s Javadoc is included. [file 1471-2105-15-150-S2.zip › additional file 2/ml2db/utils/doc/uk/ac/standrews/utils/main/stringutils/StringUtils.html]

StringUtils


JavaScript is disabled on your browser.


- Overview
- Package
- Class
- Use
- Tree
- Deprecated
- Index
- Help

- Prev Class
- Next Class

- Frames
- No Frames

- All Classes

- Summary:
- Nested |
- Field |
- Constr |
- Method

- Detail:
- Field |
- Constr |
- Method


uk.ac.standrews.utils.main.stringutils

## Class StringUtils

- java.lang.Object
- - uk.ac.standrews.utils.main.stringutils.StringUtils

- ---

    

  ```
  public class StringUtils
  extends java.lang.Object
  ```

  Class

  Version:
  :   11 Mar 2008

  Author:
  :   Luna De Ferrari luna.deferrari-at-ed.ac.uk

- - ### Constructor Summary

    Constructors

    Constructor and Description

    `StringUtils()`
  - ### Method Summary

    Methods

    | Modifier and Type | Method and Description |
    | --- | --- |
    | `static java.lang.String` | `backQuoteChars(java.lang.String string)` Converts carriage returns and new lines in a string into \r and \n. |
    | `static java.lang.String` | `booleanArrayToString(boolean[] array)` |
    | `static java.lang.String` | `booleanTo01String(java.lang.Boolean booleanValue)` Returns "1" if Boolean.TRUE, "0" if Boolean.FALSE |
    | `static boolean` | `containsPositiveInteger(java.lang.String string)` Returns true if the string contains a parsable integer |
    | `static java.lang.String` | `doubleQuote(java.lang.String text)` Enclose text in double quotes |
    | `static java.lang.String` | `doubleQuoteIfNeeded(java.lang.String text)` Enclose text in double quotes if it contains non-alphanumerical characters such as . |
    | `static java.lang.String` | `fromInputStreamToString(java.io.InputStream in)` |
    | `static java.lang.String` | `getSerialNumericalEnding(java.lang.String baseString, java.lang.String currentString)` Get a progressive +1 numerical ending. |
    | `static java.lang.String` | `intArrayToString(int[] array)` |
    | `static boolean` | `isDash(java.lang.String string)` check if the string is a dash |
    | `static java.lang.String` | `leftChopString(java.lang.String string, int characters)` Shortens the given string to the number of characters specified by chopping off characters on the left, if needed. |
    | `static java.lang.String` | `objectArrayToString(java.lang.Object[] array)` |
    | `static java.lang.String` | `quote(java.lang.String string)` From Mulan!: Quotes a string if it contains special characters. |
    | `static java.lang.String` | `rightRemoveString(java.lang.String string, java.lang.String toBeRemoved)` |
    | `static boolean` | `string_is_empty(java.lang.String string)` |
    | `static boolean` | `stringIsNullOrEmpty(java.lang.String string)` |
    | `static java.lang.String` | `stripFirstLastCharacter(java.lang.String text)` |

    - ### Methods inherited from class java.lang.Object

      `equals, getClass, hashCode, notify, notifyAll, toString, wait, wait, wait`

- - ### Constructor Detail


    - #### StringUtils

      ```
      public StringUtils()
      ```
  - ### Method Detail


    - #### string\_is\_empty

      ```
      public static boolean string_is_empty(java.lang.String string)
      ```


    - #### backQuoteChars

      ```
      public static java.lang.String backQuoteChars(java.lang.String string)
      ```

      Converts carriage returns and new lines in a string into \r and \n.
      Backquotes the following characters: ` " \ \t and %

      Parameters:
      :   `string` - the string

      Returns:
      :   the converted string

      See Also:
      :   `#unbackQuoteChars(String)`


    - #### quote

      ```
      public static java.lang.String quote(java.lang.String string)
      ```

      From Mulan!:
      Quotes a string if it contains special characters.
      The following rules are applied:
      A character is backquoted version of it is one
      of " ' % \ \n \r \t.
      A string is enclosed within single quotes if a character has been
      backquoted using the previous rule above or contains
      { } or is exactly equal to the strings
      , ? space or "" (empty string).
      A quoted question mark distinguishes it from the missing value which
      is represented as an unquoted question mark in arff files.

      Parameters:
      :   `string` - the string to be quoted

      Returns:
      :   the string (possibly quoted)

      See Also:
      :   `#unquote(String)`


    - #### booleanTo01String

      ```
      public static java.lang.String booleanTo01String(java.lang.Boolean booleanValue)
      ```

      Returns "1" if Boolean.TRUE, "0" if Boolean.FALSE

      Parameters:
      :   `booleanValue` -

      Returns:
      :   "1" if true, "0" if false


    - #### containsPositiveInteger

      ```
      public static boolean containsPositiveInteger(java.lang.String string)
      ```

      Returns true if the string contains a parsable integer


    - #### doubleQuote

      ```
      public static java.lang.String doubleQuote(java.lang.String text)
      ```

      Enclose text in double quotes

      Parameters:
      :   `text` -

      Returns:
      :   "text"


    - #### doubleQuoteIfNeeded

      ```
      public static java.lang.String doubleQuoteIfNeeded(java.lang.String text)
      ```

      Enclose text in double quotes if it contains non-alphanumerical
      characters such as . or , or -

      Parameters:
      :   `text` -

      Returns:
      :   "text" if the text contains non alpha-numerical characters, text
          otherwise


    - #### fromInputStreamToString

      ```
      public static java.lang.String fromInputStreamToString(java.io.InputStream in)
                                                      throws java.io.IOException
      ```

      Throws:
      :   `java.io.IOException`


    - #### getSerialNumericalEnding

      ```
      public static java.lang.String getSerialNumericalEnding(java.lang.String baseString,
                                              java.lang.String currentString)
      ```

      Get a progressive +1 numerical ending. Eg. if base string is "n" and
      current string is "n12", the next string will be "n13". Beware, the
      current string must be null or contain the base string + an integer.

      Parameters:
      :   `baseString` -
      :   `currentString` - if null, the returned string is "0"

      Returns:
      :   the next (+1) serial string (base string with previous numerical
          ending + 1)


    - #### isDash

      ```
      public static boolean isDash(java.lang.String string)
      ```

      check if the string is a dash


    - #### leftChopString

      ```
      public static java.lang.String leftChopString(java.lang.String string,
                                    int characters)
      ```

      Shortens the given string to the number of characters specified by
      chopping off characters on the left, if needed. Must be a string with the
      most informative part on the right...

      Parameters:
      :   `string` - a string to be chopped
      :   `characters` - the max number of characters to be left in the rightmost part
          of the string

      Returns:
      :   a string chopped from the left until its length matches the given
          number of characters


    - #### rightRemoveString

      ```
      public static java.lang.String rightRemoveString(java.lang.String string,
                                       java.lang.String toBeRemoved)
      ```


    - #### stringIsNullOrEmpty

      ```
      public static boolean stringIsNullOrEmpty(java.lang.String string)
      ```

      Parameters:
      :   `string` -

      Returns:
      :   true if a string is null or empty


    - #### stripFirstLastCharacter

      ```
      public static java.lang.String stripFirstLastCharacter(java.lang.String text)
      ```


    - #### intArrayToString

      ```
      public static java.lang.String intArrayToString(int[] array)
      ```


    - #### booleanArrayToString

      ```
      public static java.lang.String booleanArrayToString(boolean[] array)
      ```


    - #### objectArrayToString

      ```
      public static java.lang.String objectArrayToString(java.lang.Object[] array)
      ```


- Overview
- Package
- Class
- Use
- Tree
- Deprecated
- Index
- Help

- Prev Class
- Next Class

- Frames
- No Frames

- All Classes

- Summary:
- Nested |
- Field |
- Constr |
- Method

- Detail:
- Field |
- Constr |
- Method
